# Design and implementation of Metta, a metasearch engine for biomedical literature retrieval intended for systematic reviewers

**DOI:** 10.1186/2047-2501-2-1

**Published:** 2014-01-10

**Authors:** Neil R Smalheiser, Can Lin, Lifeng Jia, Yu Jiang, Aaron M Cohen, Clement Yu, John M Davis, Clive E Adams, Marian S McDonagh, Weiyi Meng

**Affiliations:** Department of Psychiatry and Psychiatric Institute, University of Illinois at Chicago, Chicago, IL 60612 USA; Department of Computer Science, Binghamton University, Binghamton, NY USA; Department of Computer Science, University of Illinois at Chicago, Chicago, IL 60612 USA; Department of Medical Informatics and Clinical Epidemiology, Oregon Health & Science University, Portland, OR USA; Division of Psychiatry, University of Nottingham, Nottingham, UK

**Keywords:** Information retrieval, Metasearch engine, Bibliometrics, Medical informatics, Evidence-based medicine, Meta-analysis, Systematic reviews

## Abstract

**Background:**

Individuals and groups who write systematic reviews and meta-analyses in evidence-based medicine regularly carry out literature searches across multiple search engines linked to different bibliographic databases, and thus have an urgent need for a suitable metasearch engine to save time spent on repeated searches and to remove duplicate publications from initial consideration. Unlike general users who generally carry out searches to find a few highly relevant (or highly recent) articles, systematic reviewers seek to obtain a comprehensive set of articles on a given topic, satisfying specific criteria. This creates special requirements and challenges for metasearch engine design and implementation.

**Methods:**

We created a federated search tool that is connected to five databases: PubMed, EMBASE, CINAHL, PsycINFO, and the Cochrane Central Register of Controlled Trials. Retrieved bibliographic records were shown online; optionally, results could be de-duplicated and exported in both BibTex and XML format.

**Results:**

The query interface was extensively modified in response to feedback from users within our team. Besides a general search track and one focused on human-related articles, we also added search tracks optimized to identify case reports and systematic reviews. Although users could modify preset search options, they were rarely if ever altered in practice. Up to several thousand retrieved records could be exported within a few minutes. De-duplication of records returned from multiple databases was carried out in a prioritized fashion that favored retaining citations returned from PubMed.

**Conclusions:**

Systematic reviewers are used to formulating complex queries using strategies and search tags that are specific for individual databases. Metta offers a different approach that may save substantial time but which requires modification of current search strategies and better indexing of randomized controlled trial articles. We envision Metta as one piece of a multi-tool pipeline that will assist systematic reviewers in retrieving, filtering and assessing publications. As such, Metta may find wide utility for anyone who is carrying out a comprehensive search of the biomedical literature.

**Electronic supplementary material:**

The online version of this article (doi:10.1186/2047-2501-2-1) contains supplementary material, which is available to authorized users.

## Background

A *metasearch engine* is a federated search tool that supports unified access to multiple search systems [1]. It contains a query interface in which a user enters a single query that is sent to multiple search engines linked to different databases; the responses returned from the search engines are gathered and merged in real time, and are displayed in a concise, organized manner. The process is deceptively simple, for a great number of technical, informatics and design issues need to be solved in order to make a practical metasearch engine. Technical issues include creating a global query interface from the query interfaces of individual search engines [2], making sure that queries are understood meaningfully for each search engine, that responses occur in a timely fashion, and that results remain correct and reliable in the face of changes and updates that may occur, independently and unpredictably, within each of the search engines or their linked databases. Informatics issues include synonym and abbreviation recognition, and other natural language processing steps intended to make queries robust and comprehensive. Design issues include making sure the interface is intuitive and easy to use, and that using the metasearch engine actually saves time and maintains performance relative to conducting searches through each search engine separately.

Individuals and groups who write systematic reviews and meta-analyses in evidence-based medicine regularly carry out literature searches in multiple bibliographic databases, and thus have an urgent need for a suitable metasearch engine to save time and to remove duplicate publications from initial consideration. However, they also have special needs which creates special requirements and challenges for metasearch engine design and implementation. Most general users may carry out searches to find a few highly relevant (or highly recent) articles, but systematic reviewers seek to obtain a comprehensive set of articles on a given topic, satisfying specific criteria [3]. The focus on high recall means that the metasearch engine needs to retrieve large numbers of bibliographic records from all search engines, and not merely the highest few from a ranked list based on relevance or recency. (Note that a record contains citation information such as author, title and journal, and may include additional fields such as abstract and database specific annotations and indexing terms). Because the indexing of articles is imperfect and not uniform across search engines, and because term usage varies considerably [4], systematic reviewers tend to create very large, complex queries that are tailored differently to take into account unique features of each search engine/database. The high cost of missing any relevant articles leads to a situation in which the initial set of retrieved records requires manual inspection and may be 10–100 times greater than the final set deemed truly relevant for consideration in a given systematic review [5].

While the process of updating systematic reviews is ongoing, and there is continuing need for systematic reviews on new topics, current resources to conduct them is constrained. One of the goals of Metta is to enable a larger group of teams with clinical, but not specialized informatics expertise, to perform systematic reviews. By moving the burden of comprehensive searching from search experts to the Metta system, the resources necessary to conduct a systematic review in a single, perhaps highly specialized, topic could be lessened. This could greatly expand the number of topics covered by systematic reviews by enabling a variety of teams with appropriate clinical expertise to conduct and publish these reviews.

In the present paper, we describe our experience in creating Metta, a metasearch engine which is intended to serve as the first step in a multi-step pipeline of informatics tools designed to reduce time and effort during the intial stages of compiling a set of relevant articles for consideration in a systematic review. Because most of the bibliographic databases have copyright and subscription restrictions, Metta is not available for use by the general public, but a working prototype for soliciting feedback and comments can be viewed at http://mengs1.cs.binghamton.edu/metta/search.action.

## Methods

Our research team is an NIH-funded multi-institutional consortium that includes computer science experts on metasearch engines (CY and WM) and their students (LJ, CL and YJ), investigators with expertise in information retrieval and data mining (NS and AC), as well as experienced systematic reviewers with backgrounds in clinical research and information science (JMD, CEA, MSM, Samantha Roberts, and Karla Soares-Weiser) who are affiliated with several major systematic review groups, namely, the Cochrane Collaboration (Schizophrenia Review Group) and the AHRQ and multi-state funded Drug Effectiveness Review Project (DERP).

We asked each of the systematic reviewers to provide a list of the most important bibliographic databases that they search in their own studies, and arrived at a consensus that five are the most important for inclusion in a metasearch engine: PubMed (which encompasses MEDLINE as well as additional records indexed in PubMed and deposited in PubMed Central); EMBASE (which overlaps extensively with MEDLINE but includes a wider range of topics in zoology and chemistry); CINAHL (which focuses on allied health fields); the Cochrane Central Register of Controlled Trials (which lists certain types of clinical trial articles); and PsycINFO (which focuses on psychology and related social science fields).

Except for PubMed [6], online access to all of these databases generally requires a subscription. (The Cochrane Library is freely available in many countries but not in most of the US). Even among leading academic medical institutions, not all have institutional subscriptions to all of these databases, and to ensure that copyrights and licenses are not violated, it is important that potential users (who may be located outside of our own research group) only have access to those databases to which they have a subscription. For the purposes of making the prototype version of Metta, for internal development by our own group, we routed users through the institutional log-in of the University of Illinois at Chicago, which has subscriptions to all databases.

## Results and discussion

**Figure 1:**
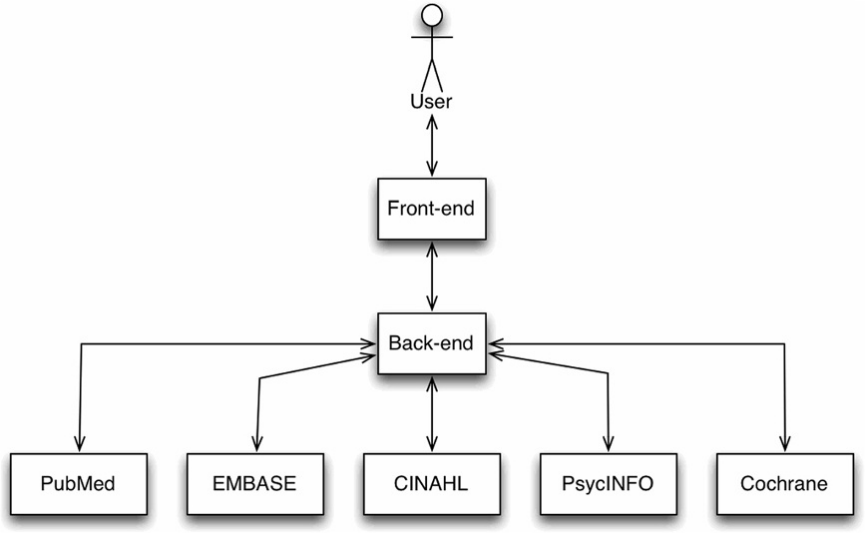
**A** **(** **very** **)** **brief summary of the metasearch engine architecture.**

As shown in Figure 1, Metta is constructed in terms of a front-end that serves as the web-based query user interface, and that interacts with a back-end that connects to the search engines to retrieve records from the 5 bibliographic databases.

### The front-end

**Figure 2:**
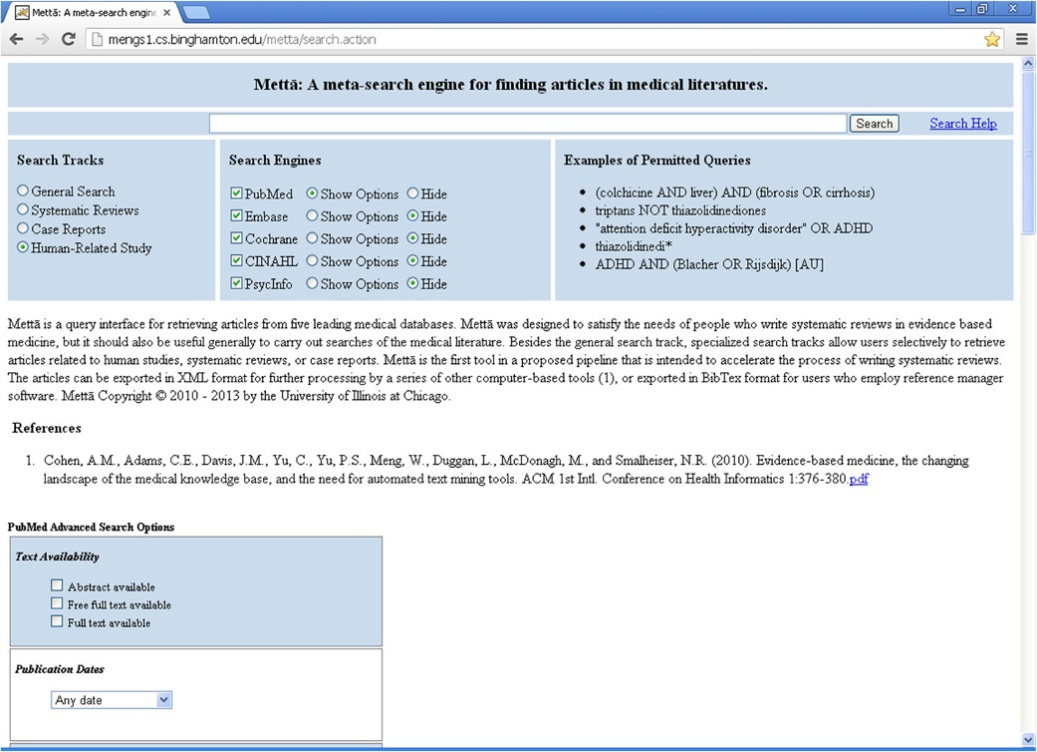
**Screenshot of the Metta home page.** A portion of the PubMed advanced search options is shown; each option can be selected or deselected by users.

In general, our aim was to simplify the process of submitting queries so that users did not need to concern themselves with complex queries, or the use of search tags or other specialized commands. On the other hand, we wanted to retain flexibility as much as possible, so that users could adjust the pre-set options for each database if they so desired. As shown in Figure 2, the Metta homepage represents a concise interface that achieved both of these aims. Users choose one of 4 different search tracks (indicated at left) to carry out either a search for human-related studies (i.e. articles which are indexed under Humans in each database); a search for case reports and similar articles; a search for systematic reviews and meta-analyses; or a general search (i.e. utilizing only the default settings of each search engine). Each search track has built-in settings designed to optimize comprehensive retrieval of the relevant types of articles, while minimizing irrelevant articles. The tailored search strategies underlying these settings are listed within the Help page (Additional file 1). Although users cannot alter the search track settings directly, this does not limit the scope of possible searches since queries can still be built up freely on the general search track. Furthermore, by clicking on “show options” for any search engine, users can manually over-ride or add options and restrictions at will, though in practice, few, if any users chose to manually adjust the search settings within individual search engine options.

In general, Metta employed the default query expansion and processing strategies of each database, with some exceptions (e.g. full-text searching was turned off for PsycINFO). Because all five databases employed three common search tags (title [ti], author [au] and abstract [ab]) these were permitted in user-entered queries. The permitted tags are shown as examples on the right side of the homepage, and in detail on the Help page (Additional file 1).

**Figure 3:**
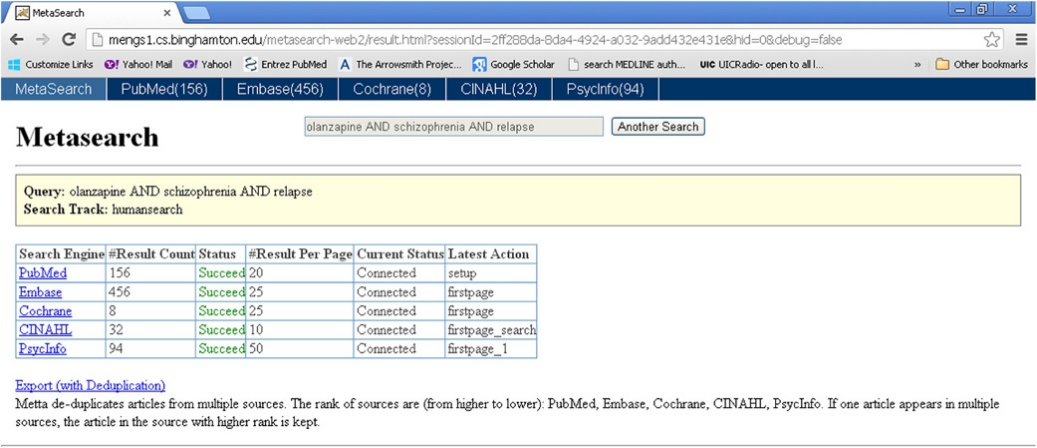
**The Metta results page.**

**Figure 4:**
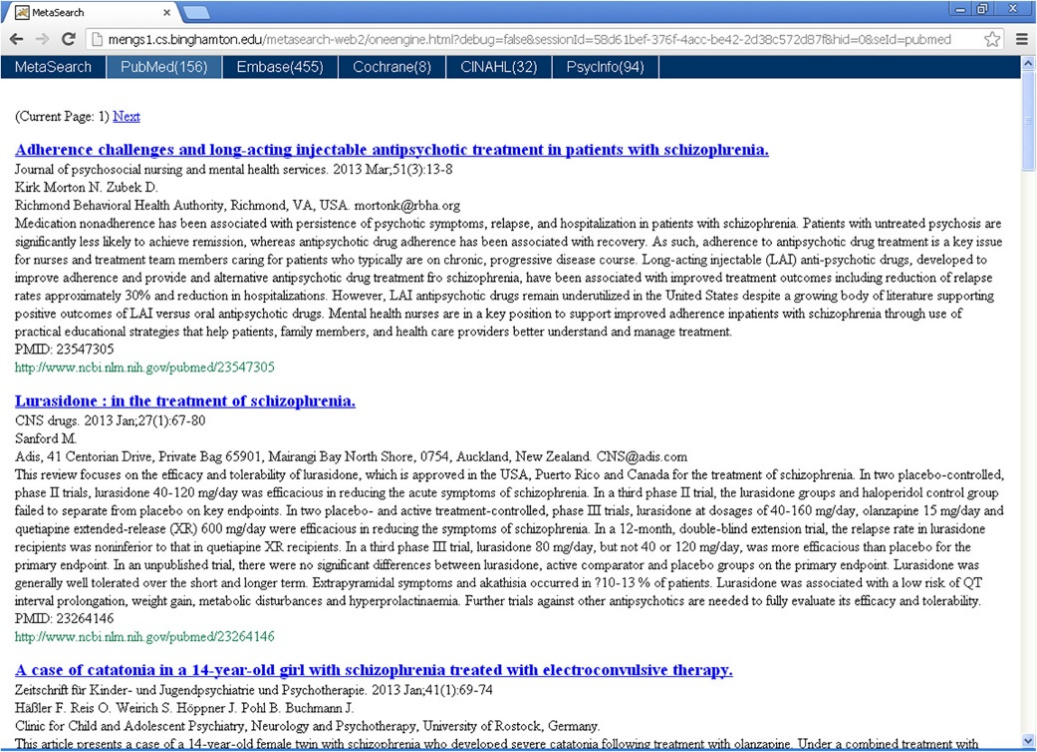
**The PubMed result page** **(** **only a portion showing** **).**

After a user inputs a query on the human track (e.g., olanzapine AND schizophrenia AND relapse) and clicks “search”, he or she will be directed to the search result page shown in Figure 3. The user inputted query and selected search track are indicated within the light yellow panel shown at the top. The query result summary is shown in the middle table, with columns showing each database with its number of results returned, connection status, and other parameter and status columns. The database name column offers the link to search result page (shown in Figure 3) of individual databases. Note that users may always switch between the result page and individual database result pages using the blue panel located at the top of the page. Figure 4 shows one of the individual database result pages, i.e. for PubMed. The snippet of each result record is in a Google-like format, showing the article title in a hyperlink to the article’s original link in the PubMed (or other source database) website, followed by journal title, publication year, issue & pages; then the authors & their affiliations; and finally abstract and original URL. Twenty records are displayed on a page.

**Figure 5:**
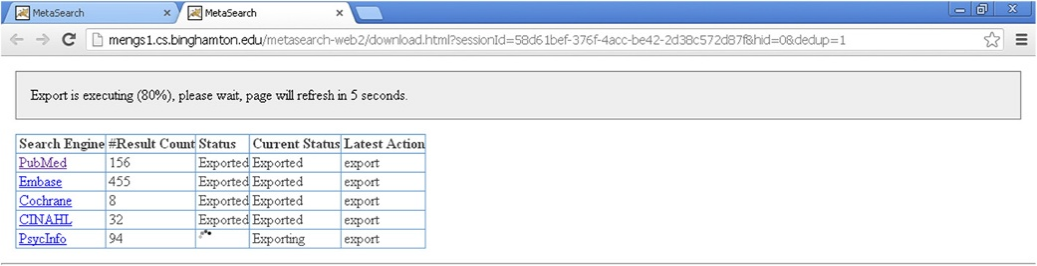
**The Metta export page** **(** **in progress** **).**

**Figure 6:**
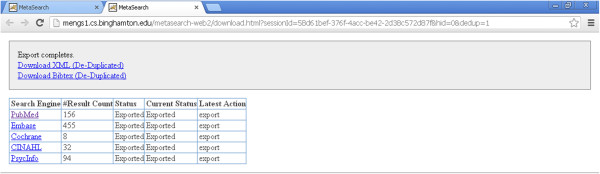
**The Metta export page** **(** **completed** **).**

On the result page (Figure 3), when the user clicks the “Export (with Deduplication)” link, this triggers Metta to pull all retrieved records from all databases, perform de-duplication for records from different databases (see below), and offer users the option to download the de-duplicated records as text files for offline use (Figures 5 and 6). The amount of time for export to be completed varies according to the number of records and their allocation among databases. Retrieval of full records was slowest for the PsycINFO database in which each record had to be re-queried and downloaded individually; although a thousand records could be exported in about a minute from the other databases, PsycINFO was at least 10 times slower. Users are offered two file download links for all the de-duplicated results, one for export in XML format (ideal for further computer processing) or BibTex format (this is designed to be compatible with with a wide range of commercial and open source reference manager software) (Figure 6).

### Design and user issues

The front-end of Metta was intended to satisfy a wide range of different types of users, as well as a wide range of different search strategies. Certainly Metta was aligned to the needs of the majority of people searching the biomedical literature, who tend to carry out only one or two queries at a time, employ only one or a few search terms, and do not routinely use search tags [7, 8]. To accommodate the needs of users who employ an iterative approach (in which initial results are examined, and the initial query modified and resubmitted), we cached queries so that all previous queries from the same session were visible as users began to type in the query box.

A much more difficult decision was how to reconcile a unified query interface with the prevailing practice of many systematic reviewers to carry out long, complex queries that involve search tags and advanced commands which are specific to individual databases. The individual databases all have optional advanced search interfaces that allow users to build up queries consisting of separate query terms (linked to field tags) concatenated with AND, OR or NOT as well as restricting search term to specific text and meta-data fields. However, entering a search tag into Metta that is inappropriate for one or more of the databases will result in query errors. We did initially implement an Advanced Search page in Metta (patterned after PubMed’s Advanced Search Builder), but finally decided to remove it and only the basic query interface is currently active.

This decision was reinforced by an analysis of the reasons that reviewers exclude initially-retrieved clinical trial articles from final inclusion in systematic reviews (discussed in detail in [9, 10]). Briefly, we found that reviewers did not trust that metadata indexing of articles was adequately reliable, particularly with regard to study design aspects (such as randomization or use of placebos), and so a) did not employ these restrictions while carrying out initial searches, and b) utilized many different word and phrase combinations in the query, in an attempt to capture all possible relevant articles. This results in an initial search that may retrieve 10–100 times more records than are finally deemed to be relevant for inclusion in the systematic review.

The resources required to conduct systematic reviews are too great for several reasons. Construction of the query is a complex process carried out by specialists, typical searches return too many articles that will be excluded from the systematic review, and doing a comprehensive search across multiple databases is time consuming and incurs a lot of manual work in deduplication and other tasks necessary to clean up the search results. Metta is only the first step in a pipeline project that creates a series of computer-assisted tools to assist systematic reviewers [11] (another step of which is to re-tag clinical trial articles with study design labels). The pipeline is intended to present an alternate manner in which systematic review literature search and initial review could be performed which reduces resource utilization in several time and resource consuming areas. We felt that it was more important to keep Metta simple and predictable in its output, rather than designing it to handle extremely large sets of retrieved records (~5,000 or more) or to facilitate the entry of extremely complex queries by users via an advanced query builder.

Thus, for systematic reviewers to be expected to adopt Metta on a routine basis, it will be necessary to re-engineer multiple steps in the process by which articles are queried, retrieved, filtered and examined for inclusion. For example, better study design annotation and filtering of articles retrieved by Metta searches should give overall high-recall retrieval with better precision than is currently obtained by conventional searches, and thus largely obviate the need of reviewers to formulate highly complex queries. In any case, one of us (NRS) has found that it is easier simply to build up complex queries on a blank Word document and then cut-and-paste it into the Metta query window, than to build up queries step by step within an advanced query page.

### The back-end

The back-end of Metta is concerned with validating permissions for each search engine; submitting queries to each search engine; ensuring that an effective connection is established and that a meaningful result is returned in real time, and presenting the results for display by the web interface (front-end), with warnings given if one or more of the data sources failed to respond. In addition, the back-end is responsible for retrieving all records regardless of the number of records displayed on each result page or other limitations of size imposed by individual search engines, and exporting retrieved records. Each of these has special requirements and will be discussed in turn.

- Validating permissions for each search engine.

The login is currently implemented in two ways: For EMBASE, it uses explicit username and password stored in the configuration file; for Cochrane, CINAHL and psycINFO, it uses the university library’s authorization to login. When it is a production system, Metta will maintain a database of users’ access rights to the search engines; every user will only be allowed to query the search engines for which they have access rights.

- b) Submitting queries to each search engine.

Each query submitted to the query interface of Metta for a selected track is mapped to a query to each of the five search engines. The mapped query for a search engine needs to satisfy the constraints of the search engine so that it can be correctly processed by the search engine. The mapping is achieved via a mapping table that keeps track of the search field correspondences between the query interface of Metta for the selected track and the query interface of each search engine, and also via information about the pre-set query options for both query interfaces of Metta for the selected track and the search engine. The mapping tables and the information about pre-set options are obtained in advance. With the exception of PubMed, mapped queries for each search engine are submitted through a connection program that is constructed based on several connection parameters extracted from the search form and website of each search engine. The basic parameters include the HTTP request method (GET or POST), search engine server name and location, and query names and pre-set options of all search fields [1]. Because PubMed’s public search interface was complex and subject to frequent changes without prior notice, we employed PubMed’s stable EUtil function for submitting and retrieving results.

- c) Ensuring that a meaningful result is returned in real time.

If Metta fails to establish connection with a search engine, or it is unable to get the search results from a search engine, this is displayed as an error on the result page. Such errors may be caused either by network connection issues or by changes within the search engine interface. Once connected, the back-end pulls and presents the results from the search engines for display by the web interface (front-end). A special issue here is that Metta must extract search result records from the response pages returned from each search engine. Response pages by search engines are dynamically generated usually by encoding data records retrieved from an underlying database into an HTML template. On the one hand, different search engines employ different templates to encode their search result records, which means that a different result record extraction program is needed for each search engine. On the other hand, each search engine usually employs a small number of templates (usually one), which makes it possible to identify the template (s) for each search engine based on sample response pages from the search engine. The key to an extraction program is a set of extraction rules (e.g., regular expressions in HTML tags) that identifies the beginning and the ending of each record from the response pages. In general, search result records extraction rules can either be generated manually, semi-automatically or automatically [1, 12, 13]. In Metta, the extraction rules for the search engines are manually created to ensure their accuracy, which is critical for the applications Metta is designed to support. It is also necessary for Metta to retrieve multiple pages of results when present, knowing how many pages there are and when the result is finished. These tasks can also be solved using rules extracted from the response pages from each search engine.

- d) Exporting retrieved records.

This includes export of full bibliographic records in XML format intended for use by automated informatics processing tools residing later in the pipeline project. We modified the XML format used by PubMed so that it was applicable across all of the databases. In addition, we created a separate link containing a text file of abbreviated bibliographic information in BibTex format, which can be readily imported into commercial reference manager software. A significant issue in this module is to identify articles that are retrieved from multiple databases. This allows duplicate results to be removed (only one copy is kept), which saves time for systematic reviewers [14]. Identification of multiple records that correspond to the same real-world entity is known as entity identification and record-linkage, and the problem has received much attention in the database community (e.g., [15, 16]). For Metta, the problem is solved in three steps. First, the semantic data units within each record are identified. For example, a citation record may have semantic data units Author, Title, Journal Name, etc. Second, a set of distance/similarity functions is used to compute how well two values of the same semantic from different records are matched. For example, an edit-distance function can be used to compute how similar two titles are. Third, for each pair of records, based on how similar their corresponding data units are, a decision is made on whether the two records are matched. Once citation records corresponding to the same article are identified, de-duplication in Metta was carried out by first retaining records that were indexed in PubMed (because of its better and more elaborate indexing scheme) and then following a priority order: EMBASE, Cochrane, CINAHL and lastly, PsycINFO. The details about the algorithms and code for de-duplication in Metta are described in a separate publication [17].

### Challenges

Metta is a working demonstration site that continues to undergo field testing and modification to serve systematic reviewers. However, a number of important challenges will be important to tackle if Metta is to become deployed as a stable production service:

a) Perhaps the biggest challenge is the fact that unannounced changes to individual search engines (and their bibliographic databases) occurred regularly, if unpredictably, every few weeks or so. Some of the programming changes were major (e.g. a website based on pages rendering relatively simple HTML changed over to Javascript). These changes could result in very subtle errors. Once detected, manual re-programming and testing were required to ensure that Metta connected and exported from each search engine accurately. To allow automatic detection of changes to the query interfaces, databases or export functions, it will be necessary to deploy a series of test scripts which run pre-specified queries (with fixed publication dates) for which the correct number of retrieved and exported records are known. These need to be run daily through each search track, and any discrepancies will be flagged for manual inspection. b) At present, errors within the system (e.g. failures of connectivity) are flagged to users. However, it will be desirable to detect and deliver error messages that arise from one or more of the search engines, e.g., when they have inputted an inappropriate query. c) It remains an open question how much query processing should be performed by Metta. Some of the individual search engines already carry out more or less extensive types of automatic query processing -- for example, PubMed employs automatic Medical Subject Heading detection and expansion, phrase lists and spelling correction. CINAHL automatically expands Boolean searches to include partial matches if no exact matches are found. One could certainly add query processing modules that automatically recognize synonyms, abbreviations and their long forms, singular vs. plural word forms, drug names, American vs. British spelling variants, and so forth. Certain standardized search strategies could be built into these query modules. For example, research building upon the Cochrane Consortium guidelines has recommended incorporating a set of query terms for retrieving randomized controlled trials [18–20], though none of these identifies 100% of the relevant articles [21, 22]. Optimized strategies for clinical queries have also long been formulated e.g., [23–25]. Such prepackaged queries could be pre-set to be selected by users with a single click. As we study further how users interact with Metta, and learn whether it is likely to gain wide acceptance from systematic reviewers (or from the general community of biomedical searchers), additional query processing modules will be added. d) At present, Metta retrieves the bibliographic records but does not show links to full text articles or pdfs that may be present. Such a feature should assist reviewers in examining the full text of potentially relevant articles.

## Conclusions

A metasearch engine for systematic reviewers is a deceptively simple endeavor, in which the technical issues are significant but the human issues are predominant. Users need to trust that a single query can effectively retrieve all relevant records from five heterogeneous databases. Each database employs different indexing schemes, and each has their own way of carrying out query processing and expansion, which constantly changes and evolves over time. Using a metasearch engine also removes some flexibility: For example, when a person carries out a search directly through PubMed, the displayed results page allows iterative filtering of the retrieved set of records, which is not possible in Metta.

Our feeling is that a metasearch engine such as Metta can play a valuable role in speeding up the process of retrieving the initial set of records during the preparation of a systematic review, as part of an overall re-engineering of the process. One option is to allow the metasearch engine stage to identify articles based on subject matter relevance, and later stages to filter these based on such aspects as publication type and study design. It is important that clinical trial articles can be comprehensively re-tagged to indicate these attributes more reliably and with better granularity, and thus overcome a major limitation of current indexing schemes. In conclusion, Metta is envisioned as comprising one component of a pipeline of informatics tools that, taken together, may re-engineer the workflow of writing systematic reviews.

## Electronic supplementary material

Additional file 1: **Contents of the Metta help page.** (DOCX 20 KB)
