# Supplementary material for: Design and implementation of Metta, a metasearch engine for biomedical literature retrieval intended for systematic reviewers
Source: Health Inf Sci Syst. 2014 Jan 10;2:1. doi: 10.1186/2047-2501-2-1 (PMC4375844; doi:10.1186/2047-2501-2-1)
Supplement: Supplementary file 1 — Additional file 1: Contents of the Metta help page. (DOCX 20 KB) [file 13755_2013_20_MOESM1_ESM.docx]

**Supplementary File 1. Contents of the Metta help page.**

| This is a guideline for composing queries for the MetaSearchEngine. |
| --- |
|  |
| A term is a content word. (e.g. diabetes). A phrase is a sequence of terms, denoting a concept (e.g. attention deficit hyperactive disorder). It is recommended that quotation marks (" ") be placed to enclose the terms within a phrase. A query can be a set of terms/phrases. For example, a query is {teenage,diabetes}. It can also be a boolean expression of terms/phrases involving the Boolean operators of AND, OR and NOT. An example Boolean query is "attention deficit hyperactivity disorder" OR ADHD. |
|  |
|  |
| **Boolean Operators** |
| **AND** : each retrieved result contains all of the terms/phrases. For example, "atypical AND antipsychotics" finds records that contain both terms/phrase. |
|  |
| **OR** : each retrieved result contains at least one of the terms/phrases. For example, "cancer OR tumor" finds results that contain either term/phrase. |
|  |
| **NOT** : excludes terms so that each retrieved result does not contain any of the terms/phrases that follow it. For example, "triptans NOT thiazolidinediones" finds results that contain the term "triptans" but not the term "thiazolidinediones" ("NOT" operator is NOT recommended). |
|  |
|  |
| To ensure that a search expression containing different operators performs as intended, parentheses are recommended. For example: (colchicine AND liver) AND (fibrosis OR cirrhosis) finds results that contain not only both "colchicine" and "liver", but also at least one of two terms, "fibrosis" and "cirrhosis". |
|  |
|  |
| **Truncation** |
| Truncation is represented by an asterisk (*). To use truncation, enter the root of a search term and replace the ending with an *. It finds all forms of that word. |
| For example, type comput* to find the words computer or computing. |
|  |
|  |
| **Search Field Tags** |
| Three types of search field tags are acceptable by the MetaSearchEngine. They are Author, Abstract and Title. When no search field tags is specified in the query, the query expression is applied to all parts of each document. |
| (Y)[AU]: Each retrieved result contains the term/phrase/subexpression Y in the author field. For example, "ADHD AND (Blacher OR Rijsdijk) [AU]" finds records that not only contain the term "ADHD", but also the author names Blacher or Pijsdijk. |
|  |
| The same principle applies to the field Abstract ([AB]) and the field Title ([TI]). |
|  |
| **Examples of Forbidden Queries** |
| (cancer AND (Smith OR James) [AU] ) [TI] |
| NOT thiazolidinediones OR triptans |
| triptans AND NOT thiazolidinediones |
|  |
|  |
| **Four Tracks in Metta** |
|  **General Search Track**: aims at generally retrieving medical literatures. |
|  **Systematic Review Track**: aims at retrieving systematic reviews. |
|  **Case Report Track**: aims at retrieving case reports. |
|  **Human-Related Study Track**: aims at retrieving human-related medical literatures. |
| Each track covers five individual search engines with their options being defaultly set. The users can modify the default options by themselves. |
| In addition, some query-composition rules (shown below) are proposed to facilitate the retrieval of type-specified literatures within Systematic Review Track and Case Report Track. |
|  |
|  General Search Track |
| The default options are set and no query-composition rules are applied to conduct the general purpose of medical literature retrieval. |
|  |
|  Systematic Review Track |
| The default options are set and the query-composition rules are proposed to ensure the retrieval of the systematic reviews. |
| **PubMed:**( Q ) AND ( "systematic review" [TIAB] OR Meta-Analysis [PT] OR systematic[sb]) |
| **Embase:**( Q ) AND ( [systematic review]/lim OR [meta analysis]/lim ) |
| **Cochrane:**No rule is applied for Cochrane within Systematic Review Track. |
| **CINAHL:**( Q ) AND ( PT "systematic review" OR PT "meta analysis") |
| **PsycInfo:** ( Q ) AND ( ti("systematic review") OR pt("meta analysis")) |
|  |
|  Case Report Track |
| The default options are set and the query-composition rules are proposed tp ensure the retrieval of case reports. |
| **PubMed:**( Q ) AND ((Case Reports [PT]) OR (English Abstract [PT] AND case[TIAB]) OR (Letter[PT] AND "case report" [TIAB]) OR (MMWR[Journal] AND (case[TIAB] OR "disease outbreaks"[MH])) OR (("journal of medical case reports"[journal] OR neurocase[journal]) AND case[TIAB] NOT review)) |
| **Embase:**( Q ) AND (("case report":ti OR "case report":ab) OR (letter:it AND ("case report":ti OR "case report":ab) ) OR ("mmwr. morbidity and mortality weekly report"/jt AND (case:ti OR case:ab)) OR (("journal of medical case reports"/jt OR "neurocase"/jt) AND (case:ti OR case:ab) NOT review)) |
| **Cochrane:**( Q ) AND "case report":pt |
| **CINAHL:**( Q ) AND ((TI "case report" OR AB "case report") OR (PT Letter AND (TI "case report" OR AB "case report")) OR (JT mmwr AND (TI case OR AB case)) OR ((SO "journal of medical case reports" OR SO neurocase)AND (TI case OR AB case) NOT review)) |
| **PsycInfo:**( Q ) AND ((ti("case report") OR ab("case report")) OR (rtype(letter) AND (ti("case report") OR ab("case report"))) OR (pub(neurocase) AND (ti(case) OR ab(case)) NOT review)) |
|  |
|  **Human-Related Study Track** |
| The default options are set to ensure the retrieval of human-related studies. |
